# Personal Oral Infection Control, Low Birthweight, and Preterm Births in Appalachia West Virginia: A Cross-Sectional Study

**DOI:** 10.1155/2018/9618507

**Published:** 2018-08-07

**Authors:** R. Constance Wiener, Christopher Waters

**Affiliations:** ^1^Assistant Professor, Dental Practice and Rural Health, School of Dentistry, Department of Epidemiology, School of Public Health, West Virginia University, P.O. Box 9448 Robert C. Byrd Health Sciences Center Addition 104a, Morgantown, WV 26506, USA; ^2^Dental Research Labs Director, 106a Health Sciences Addition, P.O. Box 9448, Department of Dental Research, School of Dentistry, West Virginia University, Morgantown, WV 26506, USA

## Abstract

**Introduction:**

Appalachia West Virginia has a higher prevalence of preterm and low birthweight babies than the US national prevalence. Many factors have been studied which are known to influence preterm births and low birthweight babies. There are limited interventions that are available to decrease the likelihood of preterm and low birthweight babies; however oral health and personal oral infection control may be helpful. The purpose of this study was to evaluate the association of limited personal oral infection control among pregnant West Virginia Appalachian women and poor birth outcomes (preterm and low birthweight babies).

**Methods:**

A secondary data analysis of data from the West Virginia Healthy Start Helping Appalachian Parents and Infants (HAPI) Project from 2005 to 2016 was conducted. The researchers determined the odds ratio of personal oral infection control with a powered toothbrush (use of the brush fewer than 13 times per week versus use of the brush 13 or more times per week) on poor birth outcomes.

**Results:**

There were 845 women who completed the oral health program within the HAPI project. In unadjusted logistic regression, women who used the powered toothbrush and brushed less frequently had greater odds of poor birth outcomes than women who brushed more frequently (odds ratio of 2.07 [1.18, 3.62]* P* = 0.011 for low birthweight babies; and an odds ratio of 1.78 [1.04, 3.02]* P* = 0.034 for preterm birth). The results remained positive but were no longer significant in adjusted analysis.

**Conclusion:**

There is a need to identify interventions that will benefit pregnant women so that their pregnancies result in healthy pregnancy outcomes.

## 1. Introduction

In 2015, there were 3.98 million births in the United States [1] and 19,805 births in West Virginia [2]. Nationally, 382,786 (9.63%) births were before 37 completed weeks of gestation (preterm births) and, in West Virginia, 2,227 (11.25%) were preterm births [2]. Nationally, there were 320,869 (8.1%) low birthweight (less than 2500 grams) births, and in West Virginia there were 1,891 (9.6%) low birthweight births [2]. Louisiana, Alabama, and West Virginia have the highest percentages of births that are preterm. Mississippi, Louisiana, Alabama, the District of Columbia, and West Virginia have the highest percentages of births that are low birthweight births [2].

There are many factors that have been associated with preterm births and low birthweight babies. Some are modifiable and some are not. Poor birth outcomes were reported to be associated with inadequate maternal gestational weight gain [3], parity, higher diastolic blood pressure [4], racism [5], other than non-Hispanic white race/ethnicity in the United States [6, 7], maternal education [8], multiple birth, smoking, alcohol consumption, gestational nutrition, caloric expenditure, prenatal care, vitamins, diabetes, maternal age, and infections (among others) [9].

However, there are other researchers who reported that they did not find similar associations for several of the factors. Maternal age and education were not reported to be factors in low birthweight in one study [4]; parental smoking was reported as a paradoxical and potentially not an exogenous factor in fetal development in another study [10]. And, maternal employment was reported as a factor in poor birth outcomes only for women working in specific occupational sectors [11].

The discussion about whether there is a relationship between low birthweight and periodontal infection during pregnancy has been ongoing for over twenty years. Offenbacher et al. [12] suggested that periodontal infection could be a possible risk factor for preterm and low birthweight babies. Several researchers have supported the association [12–16]. During pregnancy, there are increases in four major hormones (estrogen, progesterone, human chorionic gonadotropin hormone, and human chorionic somatomammotropic hormone) that can alter gingival tissue [17] making it more vulnerable to bacterial infection. Folkers et al. (1992) [18] reported 30-100% of pregnant women do have gingivitis. The body's response to bacterial infection is inflammation.

Proinflammatory cytokines are associated with gingivitis. Amniotic fluid in preterm delivery also has elevated proinflammatory markers [19]. It is the ability of periodontal pathogens to influence systemic inflammation through which pregnancy outcomes can be affected [20]. Babies born prematurely and at low birthweight are at risk for multiple health complications early and throughout life. Sixty percent of all neonatal mortality is associated with preterm birth [15]. Therefore, healthy pregnancy outcomes are significant public health concerns and improving modifiable risk factors of low birthweight and preterm births is important goals.

Personal oral infection control (brushing and flossing), regular dental examinations, and prophylaxis with dental professionals help to maintain good oral health. Good oral health has the potential to lower the risk of preterm birth and low birthweight babies. The objective of this study is to determine if the use of a power toothbrush (comparing pregnant women who use a powered toothbrush 13 or more times per week and pregnant women who use a powered toothbrush less than 13 times per week) is associated with the pregnancy outcomes of birthweight (normal, low [less than 2500 g]), and preterm birth (37 completed weeks of gestation, less than 37 completed weeks of gestation).

## 2. Methods

The data for this study were from the West Virginia Healthy Start Helping Appalachian Parents and Infants (HAPI) project from 2005 to 2016, a program funded by the Health Resources and Services Administration. The ongoing free project enrolls 400-450 women annually for prenatal and postpartum services. The counties targeted for the project are Barbour, Harrison, Marion, Monongalia, Preston, Randolph, Taylor, and Upsur counties in West Virginia. However, the project is open to any West Virginia Right from the Start participant, pregnant woman who is a West Virginia resident and has a West Virginia Medicaid card, as well as women who are postpartum/interconceptional having increased risk of poor pregnancy outcomes (women who smoke, have had postpartum depression, have a low birthweight baby, etc.) [20]. The West Virginia Right from the Start program is a statewide program for women on Medicaid or are covered by the Office of Maternal, Child, and Family Health; and, who are pregnant or have a child under age one year in which women are encouraged and prompted to make healthful choices for themselves and their children by offering home visitation services by a registered nurse or licensed social worker. The Right from the Start program is a community partner with the HAPI project.

Postpartum/interconceptional services were and continue to be available for 2 years after the baby's birth for participants in the HAPI project [20]. Some of the services included assistance and payment of transportation, childcare costs, and medication; evaluation and treatment of depression, stress, and smoking [20]. Any requests for data should be made to the HAPI administrator.

From 2005 to 2016, there were 3,930 women who were provided with prenatal HAPI services. In-home services were provided by designated care coordinators (nurses or social workers). The educational component of the services included topics on healthy pregnancies, depression, wellness, family, relationships, and infant growth and development [20]. Oral services were and continue to be available as an option to enrollees who entered the program before 28 gestation weeks. Designated care coordinators provided an initial oral health education session (supplemented with brochures and instructional materials) and reviewed important aspects of oral health and birth outcomes.

Of the 3.930 HAPI participants from 2005 to 2016, there were 203 HAPI participants who were not eligible or declined oral health services. There were 3,737 participants who received at least one oral health education session relating to oral hygiene during pregnancy. The designated care coordinators arranged oral examinations and prophylaxis for the interested women. There were 2,282 participants who attended at least the initial oral examination visit with a licensed dentist working with HAPI.

At the time of the initial examination, an additive (overall) periodontal screening and reporting score (PSR) was recorded. All teeth were examined with measurements recorded for the buccal (cheek) side of the tooth (mesially, midtooth, and distally) and on the lingual (tongue) side of the tooth (mesially, midtooth, and distally). Each tooth was coded as 0 (the colored part of the World Health organization periodontal probe was visible and there was no calculus or restoration with a defective margin); 1 (in addition to the criteria for coding as 0, there was bleeding on probing); 2 (the colored part of the periodontal probe was visible, but there was calculus or a restoration with a defective margin); 3 (the colored part of the probe was partially visible on probing); and 4 (the colored part of the probe was not visible on probing) [21]. The mouth was evaluated in sextants. Each sextant was scored with the code for the most involved tooth in that sextant. The sextant scores were combined into an additive (overall) periodontal screening and reporting score. After the examination, the women received a dental prophylaxis.

After the initial examination and prophylaxis, the designated care coordinator again met with the HAPI participant. The HAPI participant was given a Philips Sonicare^®^ toothbrush, and the designated care coordinator instructed the HAPI participant on how to use it. The HAPI participant was also given a month's supply of toothpaste. Each month after that visit, they received additional toothpaste.

After the HAPI participant delivered her baby, she was invited to return to her dentist for a follow-up visit. A second examination, with PSR, and prophylaxis were completed. There were 891 (39.0%) who completed the second PSR.

This particular study is a secondary data analysis of HAPI data collected from 2005 to 2016. The criteria for inclusion into this study were that the women were participants in the HAPI project (that is, West Virginia residency, Medicaid, or Office of Maternal, Child, and Family Health coverage, and pregnancy), completed the oral health education portion of the HAPI program, completed the first dental visit (examination, PSR, and prophylaxis), received a power toothbrush, were delivered a singleton baby, and completed the second dental visit (examination, PSR, and prophylaxis). Otherwise, enrolled women were not excluded.

The variables of interest for this study were tooth brushing frequency, which we termed personal oral infection control (13 or more times per week, less than 13 times per week) with the power toothbrush. The American Dental Association recommends brushing twice daily; therefore, the use of less than 13 and 13 or more were chosen as the cut-points for the study. Two birth outcomes were of interest: preterm births (37 completed weeks of gestation, or less than 37 completed weeks of gestation) and birthweight (2500g, or less than 2500 g).

### 2.1. Statistical Analyses

Data analyses included frequency determinations, Chi square test, and logistic regression analyses. Inclusion criteria were that the participant completed all oral health aspects of the project, the participant had a singleton birth, and data were available on personal oral infection control, birthweight, and completed weeks of gestation.

An a priori alpha of 0.05 was established for the statistical tests. Variables with a 90%-10% split or similar indication of a nearly homogenous group were not to be included in adjusted analyses (therefore sex, income [all participants were on Medicaid or had coverage through the Office of Maternal, Child and Family Health], insurance, race/ethnicity, and education were not included).

Variables of interest, but not available in the dataset, were systemic disease/Charlson comorbidity index; specific diseases such as diabetes; maternal age, education, race/ethnicity, and income. As the participants are from West Virginia counties in which there are from 91.0% to 99.5% non-Hispanic white residents and these percentages are representative of the statewide population (93.9% non-Hispanic white) [22], the race/ethnicity of the participants were considered homogenous and fit the requirement to be excluded in the adjusted analyses.

There were 81.0% to 91.6% of the residents over age 25 years in the target counties who had a high school degree or above. This number was also representative of the statewide population in which 85% of people over age 25 years had a high school degree or above [22]. Although such data were not available in the dataset, the HAPI participants were considered to be homogenous on this category as well.

### 2.2. Ethics Approval

The study was acknowledged as nonhuman subject research (secondary data analysis) by the West Virginia University Institutional Review Board (Protocol 1702467856) and follows the strengthening the reporting of observational studies in epidemiology (STROBE) guidelines.

## 3. Results

Of the 3,930 women who participated in the HAPI project prenatal services program and 2,282 who had an initial oral evaluation with a dentist, 891 completed all oral health aspects of the project (39.0%). There were 845 who had singleton births (46 women, 5.2% had twins).

The mean of completed weeks of gestation was 38.7 weeks (Standard Deviation [SD]: 2.38 weeks). The mean birthweight was 3,320 grams (SD, 591.6 grams). There were 59 (7.0%) babies who were preterm, 53 (6.3%) who were low birthweight, and 20 (2.4%) who were very low birthweight.

During pregnancy, there were 19 (2.2%) women who reported no personal oral infection control. Sixty-five women reported personal oral infection control 1-5 times per week. There were 222 who reported personal oral infection control 6-12 times per week, and 539 reported personal oral infection control 13 or more times per week. (Table 1).

There was a significant difference between mothers whose personal oral infection control was less frequent than 13 times per week and mothers whose personal oral infection control was 13 or more times per week in terms of preterm births (*P* = .024) in the Fisher Exact test. Mothers whose personal oral infection control was less frequent than 13 times per week were more likely to have preterm births. Similarly, there was a significant difference between mothers whose personal oral infection control was less frequent than 13 times per week and mothers whose personal oral infection control was 13 or more times per week in terms of having a baby with low birthweight (*P* = .008) in the Fisher Exact test. Mothers whose personal oral infection control was less frequent than 13 times per week were more likely to have babies with low birthweight (Table 2).

In unadjusted logistic regression on low birthweight, mothers whose personal oral infection control was less than 13 times per week had an odds ratio of 2.07 (95% Confidence Interval [CI]: 1.18, 3.62;* P* = 0.011) as compared with mothers whose personal oral infection control was 13 or more times per week. In adjusted regression, the odds ratio was 1.82 (95% CI: 0.96, 3.47;* P* = 0.068) (Table 3).

In unadjusted logistic regression on preterm birth, mothers whose personal oral infection control was less than 13 times per week had an odds ratio of 1.78 (95% CI: 1.04, 3.02;* P* = 0.034) as compared with mothers whose personal oral infection control was 13 or more times per week. In adjusted regression, the odds ratio was 1.31 (95% CI: 0.70, 2.43;* P* = 0.403) (Table 4).

## 4. Discussion

In this study of personal oral infection control and pregnancy outcomes of women in Appalachia West Virginia, women who maintained personal oral infection control with a power toothbrush at levels of 13 or more times per week were more likely to deliver at term and to have babies of normal weight than women who did not use the powered toothbrushes at 13 or more times per week in the unadjusted analyses. The association was significant in the bivariate analyses and in unadjusted logistic regression. The association remained positive but was no longer statistically significant with the addition of other factors in adjusted logistic regression.

There are few similar studies with which to compare this study. Researchers who conducted a literature review of 7,754 articles from five search engines found that there were few studies with maternal interventions for oral health wellness, hygiene behaviors, and oral systemic associations [23]. In our search of the literature of similar studies examining the benefits of power toothbrush use during pregnancy (PubMed, Google Scholar, and EbscoHost), there were only two peer-reviewed articles that were similar in focus with this study. Researchers in North Carolina had an intervention group of 35 pregnant women who received subgingival scaling and root planing (SRP) and a sonic toothbrush, as well as a control group of 32 pregnant women who received a delayed supragingival debridement and manual toothbrush during pregnancy followed by postpartum SRP and delivery of a sonic toothbrush [24]. The researchers found that the early prenatal SRP and homecare with the sonic toothbrush had a 3.8-fold decrease in the rate of preterm delivery [24].

Researchers involving 120 pregnant women in Alabama provided nonsurgical therapy at baseline and oral hygiene products, including a power toothbrush [19]. Ninety completed the program and 30 did not complete the program. The researchers found a preterm birth rate of 6.7% and a low birthweight birth rate of 10.2% among the women who completed the program. The results failed to reach a significant difference between the women who received the oral intervention and women who did not complete the program [19]. However, these researchers noted that Gram-negative bacterial infections may initiate cell-mediated immune responses which increase cytokines (Interleukin-1 beta, Interleuken-6, and tumor necrosis factor alpha) and prostaglandins which may lead to preterm births and low birthweight babies; therefore there is a need for additional studies with control groups [19].

The current study, involving personal oral infection control self-care with a powered toothbrush, was part of the HAPI service program to minimize preterm births and low birthweight babies and help women attain their own health. The overall success of the HAPI program should be noted. Women who participated in the HAPI program had a preterm birth rate of 7.0%, which was below the national rate of 9.3% and well below the West Virginia rate of 11.25%. Similarly, the low birthweight rate for the women who participated in the HAPI program was 6.3%, which was below the national rate of 8.1% and well below the West Virginia rate of 9.6%.

This study has strengths and limitations. A strength is that the study is a multiyear study. However, a limitation is that although it is a multiyear study, the sample size may not be large enough to identify differences between the groups with the addition of other factors which also influence gestation in multivariable analyses. For example, the* P* value for low birthweight in the adjusted logistic regression model was 0.068. Although the relationship remained positive for low birthweight and less frequent personal oral infection control in the multivariable logistic regression model, a larger sample size may have resulted in a significant association of low birthweight and infrequent brushing.

This study sample was considered to be very homogenous and, as such, the homogenous variables were not included in the multivariable logistic regression analyses. The participants were homogenous in that all participants were, of course, women, and all were on Medicaid or had coverage through the Office of Maternal, Child, and Family Health, indicating that all had insurance coverage and also indicating that they had low income to be able to participate in such programs. All had received oral health education, evaluation, power tooth brushes, and the support of the designated care coordinators to help them have healthy babies. All of the participants were from West Virginia counties in which there were from 91.0% to 99.5% non-Hispanic white residents and in which 81.0% to 91.6% of residents over 25 years had a high school education or above [22]; therefore the race/ethnicity and educational level of the participants were considered homogenous. Nevertheless, these data were not available from the dataset. Additionally, data concerning systemic disease/Charlson comorbidity index, smoking, and specific diseases such as diabetes were not available.

As an observational study, rather than interventional study, this study, by nature, does not have a control group and causality cannot be determined as a result. However, studies of pregnant women, a vulnerable population, have ethical considerations in research and observational studies provide much needed knowledge concerning pregnancy outcomes.

This study is semiecological in that the women were from the same culture, same geographical area, and same race/ethnicity. Having a homogeneous group is a benefit in understanding the specific group, but the results may not be generalizable to other populations.

## 5. Conclusion

There is a need to identify interventions that will benefit pregnant women so that their pregnancies result in healthy pregnancy outcomes. Further research is needed toward that end in terms of oral health interventions, particularly with high-risk individuals.

## Figures and Tables

**Table 1:** Sample characteristics, N = 845. Mean (Standard Deviation) or frequency (%).

**Baby Characteristics**	
Gestation	38.7 weeks (2.38)
Birthweight	3320.1 grams (591.6)
Preterm	59 (7.0%)
Birthweight below 2500 grams	53 (6.3%)
Birthweight below 2000 grams	20 (2.4%)

**Mother's Daily Oral Infection Control/Week**	

None	19 (2.2%)
1-5 times/week	65 (7.7%)
6-12 times/week	222 (26.3%)
13 or more times/week	539 (63.8%)

**Additive Periodontal Screening and Reporting Sextant Score**	

Mean Score	11.1 (5.1%)

**Table 2:** Low birthweight and preterm birth by personal oral infection control/week.

Personal Oral Infection Control			
	Less than 13 times per week	13 or more times per week	Fisher's Exact *P*-value (1 sided)
**Preterm**			.024
Yes	29	30	
No	277	509	

**Low birthweight**			.008
Yes	28	25	
No	278	514	

Low birthweight, defined as less than 2500 grams.

Preterm, defined as less than 37 weeks gestation.

**Table 3:** Logistic regressions on low birthweight.

	Unadjusted Odds Ratio [95% CI]	*P*-value	Adjusted Odds Ratio [95% CI]	*P*-value
**Daily oral infection control**				
Less than 13 times per week	2.07 [1.18,3.62]	0.011	1.82 [0.96,3.47]	0.068
13 or more times per week	reference (1.00)		reference (1.00)	

*Abbreviation.* CI: confidence interval.

Low birthweight, defined as less than 2500 grams.

Adjusted for preterm birth and the additive periodontal screening and reporting score of the sextants.

**Table 4:** Logistic regressions on preterm birth.

	Unadjusted Odds Ratio [95% CI]	*P* value	Adjusted Odds Ratio 95% [CI]	*P* value
**Daily oral infection control**				
Less than 13 times per week	1.78 [1.04,3.02]	0.034	1.31 [0.70,2.43]	0.403
13 or more times per week	reference (1.00)		reference (1.00)	

*Abbreviation.* CI: confidence interval.

Preterm, defined as less than 37 weeks gestation

Adjusted for low birthweight and the additive periodontal screening and reporting score of the sextants.

## Data Availability

The data used to support the findings of this study are available from the corresponding author upon request.
